# Crosstalk between oxidative stress and neutrophil response in early ischemic stroke: a comprehensive transcriptome analysis

**DOI:** 10.3389/fimmu.2023.1134956

**Published:** 2023-04-26

**Authors:** Changqing Mu, Yanzhi Wang, Chen Han, Hui Song, Qian Wu, Junyi Yang, Na Guo, Yumei Ma, Chenguang Zhang, Jian Zhang, Xu Liu

**Affiliations:** ^1^ Department of Neurology, First Affiliated Hospital of China Medical University, Shenyang, Liaoning, China; ^2^ Department of Cell Biology, Key Laboratory of Cell Biology, Ministry of Public Health, Shenyang, Liaoning, China; ^3^ Key Laboratory of Medical Cell Biology, Ministry of Education, China Medical University, Shenyang, Liaoning, China

**Keywords:** ischemic stroke, oxidative stress, neutrophil response, neutrophil extracellular trap, bioinformatics analysis, weighted gene co-expression network analysis

## Abstract

**Background:**

Ischemic stroke (IS) is the second leading cause of mortality worldwide, continuing to be a serious health concern. It is well known that oxidative stress and neutrophil response play vital roles in the pathophysiology of early IS. However, the complex interactions and critical genes associated with them have not been fully understood.

**Methods:**

Two datasets (GSE37587 and GSE16561) from the Gene Expression Omnibus database were extracted and integrated as the discovery dataset. Subsequent GSVA and WGCNA approaches were used to investigate IS-specific oxidative stress-related genes (ISOSGS). Then, we explored IS-specific neutrophil-associated genes (ISNGS) using CIBERSORT analysis. Next, the protein-protein interaction network was established to ascertain candidate critical genes related with oxidative stress and neutrophil response. Furthermore, these candidate genes were validated using GSE58294 dataset and our clinical samples by RT-qPCR method. Finally, functional annotation, diagnostic capability evaluation and drug-gene interactions were performed by using GSEA analysis, ROC curves and DGIDB database.

**Result:**

In our analysis of discovery dataset, 155 genes were determined as ISOSGS and 559 genes were defined as ISNGS. Afterward, 9 candidate genes were identified through the intersection of ISOSGS and ISNGS, PPI network construction, and filtration by degree algorithm. Then, six real critical genes, including STAT3, MMP9, AQP9, SELL, FPR1, and IRAK3, passed the validation using the GSE58294 dataset and our clinical samples. Further functional annotation analysis indicated these critical genes were associated with neutrophil response, especially neutrophil extracellular trap. Meanwhile, they had a good diagnostic performance. Lastly, 53 potential drugs targeting these genes were predicted by DGIDB database.

**Conclusion:**

We identified 6 critical genes, STAT3, FPR1, AQP9, SELL, MMP9 and IRAK3, related to oxidative stress and neutrophil response in early IS, which may provide new insights into understanding the pathophysiological mechanism of IS. We hope our analysis could help develop novel diagnostic biomarkers and therapeutic strategies for IS.

## 1 Introduction

As a devastating neurological disease, ischemic stroke (IS) is a major cause of death and adult disability worldwide, thus imposing a substantial socioeconomic burden (1). Globally, nearly 7.6 million patients suffered from IS in 2019, and the incidence of IS is increasing progressively year by year (2). In the United States alone, total IS-related costs were estimated at $12.6 billion in 2012 and are expected to climb up to $241 billion by 2030 (3, 4). Thus, an increasing number of studies have been performed to explore potential drugs for IS treatment. However, till now, recombinant tissue plasminogen activator (rtPA) remains the only effective drug authorized by FDA. Meanwhile, due to a narrow therapeutic window, only 5% of IS patients benefit from rtPA (5). Therefore, there is an urgent need to explore the underlying pathophysiological mechanisms of early IS in order to find possible therapeutic targets.

During the initiation and progression of early IS, excessive oxidative stress is generated due to cerebral ischemia-reperfusion (I/R) process (6). These detrimental reactive oxygen species (ROS) could cause endothelial injury and abnormal neuron death, exacerbate subsequent neurological deficits and even lead to individual death. In addition, peripheral inflammation has been considered as another important participant in early IS. As the first leukocyte subset infiltrating the ischemic brain, neutrophils can cross injured endothelium and release various pro-inflammatory mediators to activate microglia, aggravating neuroinflammation following cerebral ischemia (7). Then, the microglia would destroy the blood-brain barrier (BBB) and subsequently recruit more activated neutrophils to migrate from peripheral blood to the ischemic brain tissue. In addition to the vicious circle between the peripheral and central inflammation, neutrophils could also form neutrophil extracellular traps (NETs), which have been shown to promote cerebral thrombosis and brain I/R damage in early IS (8). However, the critical genes related with oxidative stress and neutrophil response are still unclear and deserve further study.

In recent years, transcriptomic bioinformatics has been used to investigate the molecular mechanism of various human diseases, showing great promise in helping researchers deepen the understanding of disease etiology and explore potential therapeutic targets (9). Previous bioinformatics analyses have already found several hub genes that play important roles in stroke pathogenesis (10, 11). However, to our knowledge, no studies have identified the IS-related critical genes involved in both oxidative stress and neutrophil responses simultaneously. Hence, in this study, we first used GSE16561 and GSE37587 as discovery datasets to identify candidate critical genes associated with oxidative stress and neutrophil response in early IS. Then, six real critical genes (STAT3, MMP9, AQP9, SELL, FPR1, and IRAK3) were further validated in another dataset GSE58294 and our clinical samples using reverse transcription-quantitative polymerase chain reaction (RT-qPCR) method. Subsequent functional annotation analysis showed these 6 critical genes were related with neutrophil response, including neutrophil extracellular trap. Moreover, the 6 critical genes had a good diagnostic performance for IS. Lastly, we predicted 53 potential drugs that may exert neuroprotective effects in early IS by targeting these genes. We hope our study could provide new enlightenment for individualized diagnosis and treatment of IS.

## 2 Material and methods

### 2.1 Data selection and description

The GEO database (http://www.ncbi.nlm.nih.gov/geo) was used to search the term “ischemic stroke” for early ischemic stroke (IS) gene expression profiles. The criteria for filtering the obtained datasets were as follows: (i) expression profile type is microarray data containing genome-wide mRNA expression, (ii) each dataset includes at least 20 IS patient samples, (iii) whole blood samples are collected within 48 hours from known onset of symptom. Finally, we selected the datasets of GSE37587, GSE16561, and GSE58294. The details of the datasets were listed in **Table S1**.

### 2.2 Data preparation and study design

**Figure 1:**
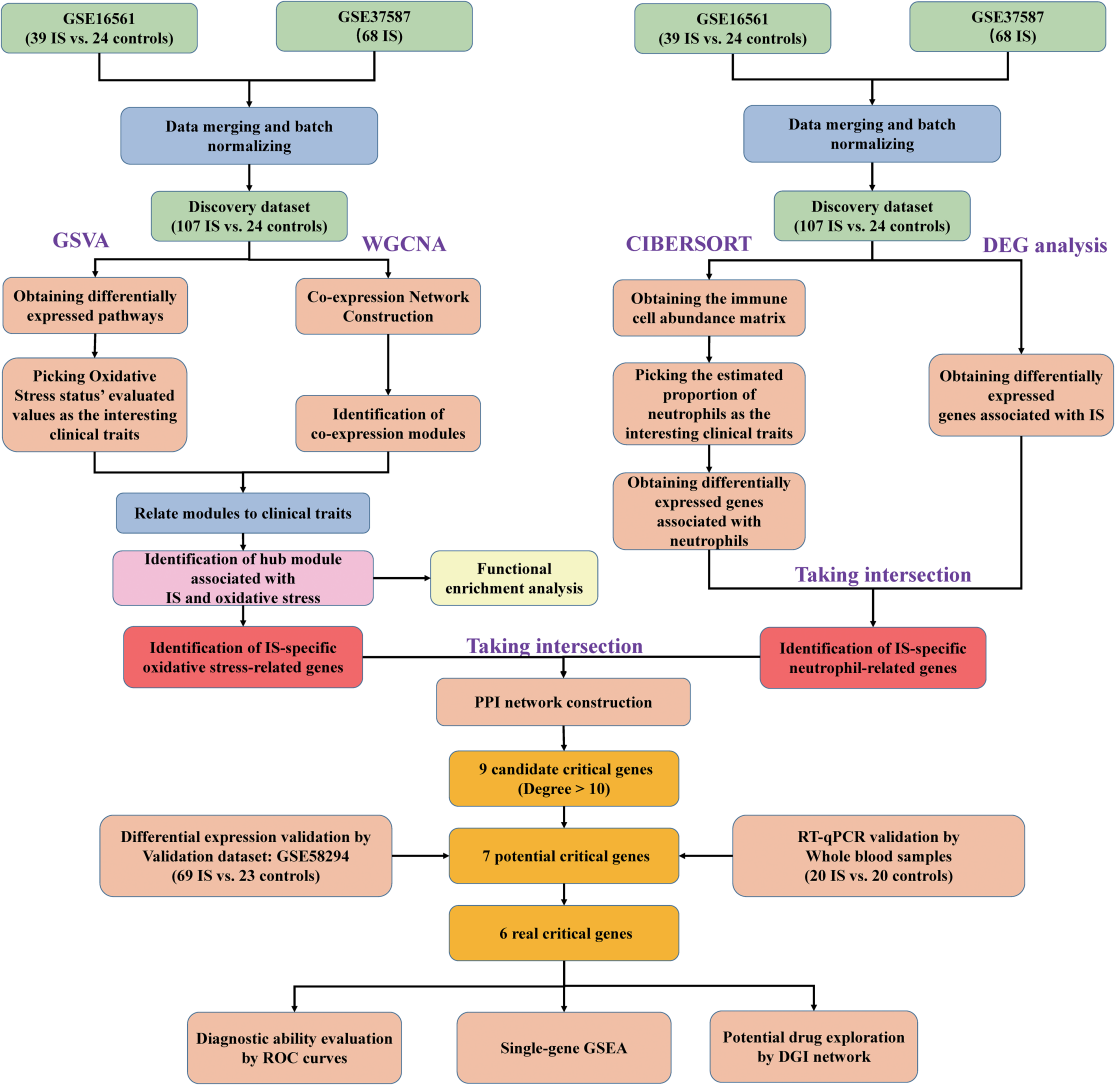
Flow chart of the transcriptomic bioinformatics analysis for early ischemic stroke.

The following bioinformatics analysis was conducted with R software (version 4.0.5). The background correction, normalization and log2-transformation were performed on the data of the three datasets. ID conversion was subsequently conducted in line with the probe annotation information. Then, eliminating the batch effects *via* ComBat in “sva” package, GSE37587 and GSE16561 were integrated into a large expression matrix as the discovery dataset. In addition, the principal component analysis (PCA) was performed to test the quality of the merged data. The flow diagram of the comprehensive analysis is shown in **Figure 1**.

### 2.3 Identification and function annotation of IS-specific oxidative stress-related gene set

The oxidative stress gene set of WikiPathways subset of Canonical pathways was obtained from the Molecular Signatures Data base (MSigDB) (https://www.gsea-msigdb.org/gsea/msigdb/). First, the enrichment scores of each sample from the discovery dataset were computed by the gsva algorithm and “gsva” package. Second, the Bayesian algorithm in the “limma” package was used to find differential pathways between IS and controls. Third, using the “wgcna” package, a scale-free co-expression network of the genes with the top 25% variance was established and the IS-specific oxidative stress-related module was defined. Finally, in the selected module, the genes with |gene significance (GS) value| for IS> 0.2, |GS value| for oxidative stress > 0.2 and |Module Membership (MM) value| > 0.8 were determined as the IS-specific oxidative stress-related gene set (ISOSGS). Furthermore, the “ClusterProfiler” package was employed to identify enriched function annotation of ISOSGS, which included Gene Ontology (GO) terms consisting of biological processes (BP), cellular components (CC), and molecular function (MF) as well as the Kyoto Encyclopedia of Genes and Genomes (KEGG) pathways.

### 2.4 Identification of IS-specific neutrophil-related gene set

CIBERSORT deconvolution algorithm was applied to estimate the abundance of 22 types of infiltrated immune cells among 131 samples of the discovery dataset. Afterward, comparing neutrophil-high group with neutrophil-low group based on the estimated fractions, genes with |log2 fold change (FC)| > 0.5 and P < 0.05 were identified as neutrophil-related differentially expressed genes (DEGs) by the “limma” package. Likewise, IS-specific DEGs between IS and controls were achieved. Ultimately, the overlapped genes between neutrophil-related DEGs and IS-specific DEGs were considered as IS-specific neutrophil-related gene set (ISNGS).

### 2.5 Ascertainment of critical genes associated with oxidative stress and neutrophil response

A protein-protein interaction (PPI) network of the genes taking the intersection of the ISOSGS and ISNGS was further constructed using the Search Tool for the Retrieval of Interacting Genes (STRING) database (https://string-db.org/). For PPI construction, seven active interaction sources (text-mining, experiments, databases, co-expression, neighborhood, gene fusion and co−occurrence) were used, and only nodes of query proteins with confidence score > 0.15 were enrolled in the network. Then, the established network was imported into Cytoscape software (version 3.8.2) and the genes were ranked by degree algorithm using the plugin cytohubba. Lastly, the genes of PPI network with degree > 10 were screened as candidate critical genes.

### 2.6 Dataset and clinical samples validation for candidate critical genes

The GSE58294 dataset was set as replication cohort and analyzed for verifying the expression differences of candidate critical genes. All samples were dichotomized into neutrophil-high and neutrophil-low groups, as well as oxidative stress-high and oxidative stress-low groups by the median of neutrophil proportions using CIBERSORT and oxidative stress scores by GSVA, respectively. Subsequently, the expression differences of candidate critical genes were compared between IS and controls, neutrophil-high and neutrophil-low groups, oxidative stress-high and oxidative stress-low groups using t-test.

To further verify the differential expression of candidate critical genes, another case-control gene expression analysis involving 20 IS patients and 20 controls was performed using whole blood samples. The clinical characteristics of these samples were listed in **Table S2**. This study was approved by the ethical committee of The First Affiliated Hospital, China Medical University. Informed consent was obtained from all participating individuals.

Specifically, peripheral blood samples were collected in EDTA-coated blood tubes, and samples were immediately pretreated, including plasma depletion, RBC lysis and addition of TRIzol reagent (Invitrogen, USA). The time from sample collection to RNA extraction was no more than 2 hours. Subsequently, total RNA was extracted, reverse-transcribed into cDNA, and stored at -80°C until use. The Takara PrimeScript RT Master Mix and SYBR Green Premix were used in the reverse transcriptase reaction and PCR amplification, respectively. The PCR primer sequences applied in the experiment are shown in **Table S3**. All samples were examined in triplicate. The PCR validation results were quantified through the 2-ΔΔCt method (livak method) with the normalization to GAPDH.

Lastly, candidate genes replicated with the GSE58294 dataset and further validated by our clinical samples were identified as real critical genes.

### 2.7 Functional annotation and diagnostic capability evaluation for critical genes

For assessing the activation/suppression of signaling pathways associated with critical genes in early IS, a gene set enrichment analysis (GSEA) based on KEGG pathways in the MSigDB database was conducted using the “ClusterProfiler” package. The pathways with |normalized enrichment score (NES)| > 2 and q value < 0.05 were considered as significantly activated/suppressed. Additionally, to evaluate the diagnostic power of critical genes for early IS, receiver operating characteristic (ROC) curves and areas under the curve (AUC) were calculated and plotted by the “pROC” package. AUC > 0.7 was considered to be a good indicator of diagnostic performance.

### 2.8 Exploration of potential drugs targeting critical genes

The Drug-Gene Interaction Database (DGIDB, http://www.dgidb.org/) is a web resource integrating drug-gene interactions and druggability data. For exploring potential therapeutic opportunities, a drug-gene network of critical genes was constructed using the drug-gene interactions predicted by DGIDB (version 4.2.0). The drug-gene interaction network was visualized by using Cytoscape software.

### 2.9 Statistical analysis

Continuous variables were summarized as mean ± SD and categorical variables as numbers (percentages). Normality distribution of continuous variables was tested with the Shapiro-Wilk test. Differences of continuous variables between groups were evaluated by the Student’s *t* test. Categorical variables were compared by Chi-square test or *Fisher*’s exact test. A two-sided *P* value of 0.05 was considered statistically significant. Statistical analyses were performed using R (version 4.0.5) and GraphPad Prism 8 (GraphPad Software, Inc).

## 3 Results

### 3.1 Data preprocessing

After background correction, normalization, log2-transformation, ID conversion and batch calibration, the discovery dataset was merged by GSE37587 (containing 68 early IS) and GSE16561 (containing 39 early IS and 24 healthy controls). Then, we performed a PCA analysis and used the PCA scatter diagrams to show the results before and after batch correction. We found that samples from two different datasets were obviously distributed in two non-adjacent areas before data integration, but almost concentrated in the same area after removing the batch effect, demonstrating the data reliability of the discovery dataset (**Figure S1**).

### 3.2 Identification of ISOSGS

**Figure 2:**
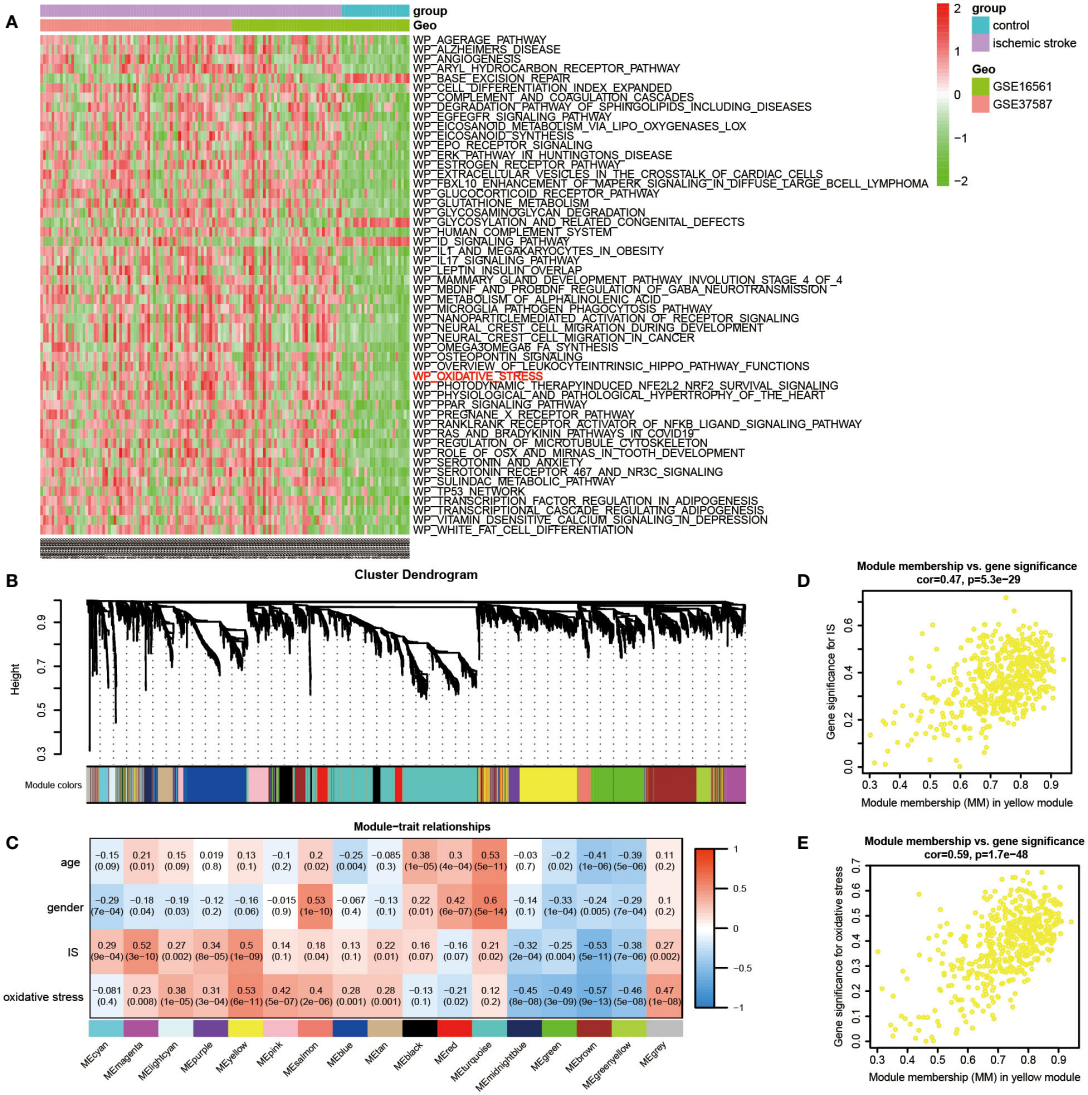
Identification of IS-specific oxidative stress-related genes (ISOSGS). **(A)** Heatmap of differential pathways showing significantly up-regulated oxidative stress pathway in early IS. **(B)** Cluster dendrogram of 17 gene modules. **(C)** The heatmap of module-trait relationships. **(D)** Scatter diagrams of module membership *vs*. gene significance for IS in yellow module; **(E)** Scatter diagrams of module membership *vs*. gene significance for oxidative stress state in yellow module.

First, following the data preparation, the changes in each pathway activity in early IS and controls of the discovery dataset were evaluated through gene set variation analysis (GSVA) using WikiPathways (664 gene sets) from MSigDB. At thresholds of adjusted P value < 1E-05 and |log2 FC| > 0.3, 52 significantly differential pathways were identified (49 up-regulated and 3 down-regulated). As shown in the heatmap, the oxidative stress pathway is included among the top up-regulated pathways, implying the importance of oxidative stress in early IS (**Figure 2A**). In the subsequent analysis, we set GSVA scores of oxidative stress as one of the sample traits and introduced WGCNA to explore the potential genes associated with both IS and oxidative stress. Initially, a total of 4, 516 genes with the top 25% variance of discovery dataset were involved in the analysis. Then, no outliers were detected after hierarchical clustering of all samples (**Figure S2A**). Next, under scale-free R2 > 0.85, a minimal beta value of 7 was chosen as the soft-threshold power (**Figures S2B** and **2C**). Consequently, WGCNA identified 17 co-expression modules (**Figure 2B**). The relationships of these modules with sample traits (IS, age, gender and oxidative stress) were demonstrated in the correlation heatmap (**Figure 2C**). The yellow module among 17 modules showed statistically significant and highest correlations with IS (Pearson co-efficient = 0.5, P = 1E-09) and oxidative stress (Pearson co-efficient = 0.53, P = 6E-11), but not age (P = 0.1) and gender (P = 0.06). Thus, we defined yellow module as the IS-specific oxidative stress-related module. Furthermore, **Figures 2D**, **E** displayed the correlations between the MM value for each gene in the yellow module and the corresponding GS value for IS as well as oxidative stress, respectively. Finally, in the yellow module (with 503 genes), 155 genes with |GS value for IS| > 0.2, |GS value for oxidative stress| > 0.2 and |MM value| > 0.8 were ascertained as ISOSGS.

### 3.3 Functional enrichment analysis of ISOSGS

**Figure 3:**
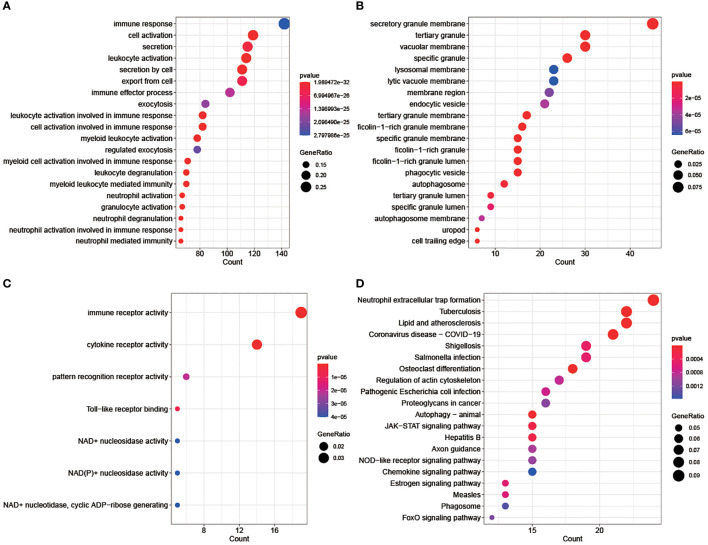
Bubble diagrams displaying the top 20 significant enrichment terms for the ISOSGS. **(A)** BP terms; **(B)** CC terms; **(C)** MF terms; **(D)** KEGG terms.

GO and KEGG analysis further investigated the potential biological functions involved in ISOSGS, where the enrichment results for BP, CC, and MF terms as well as KEGG pathways were exhibited in the bubble plots. Interestingly, BP enrichment of ISOSGS were found associated with immune response, immune effector process, myeloid leukocyte activation, myeloid leukocyte mediated immunity, myeloid cell activation involved in immune response, leukocyte activation, leukocyte degranulation, leukocyte activation involved in immune response, neutrophil activation, neutrophil degranulation, neutrophil mediated immunity and neutrophil activation involved in immune response (**Figure 3A**). Meanwhile, the most significant CC and MF were secretory granule membrane and immune receptor activity, respectively (**Figures 3B, C**). Besides, for the KEGG pathways, ISOSGS were mainly enriched in the following signaling pathways including neutrophil extracellular trap formation, chemokine signaling pathway, autophagy, FoxO signaling pathway and endocytosis (**Figure 3D**).

### 3.4 Identification of ISNGS

**Figure 4:**
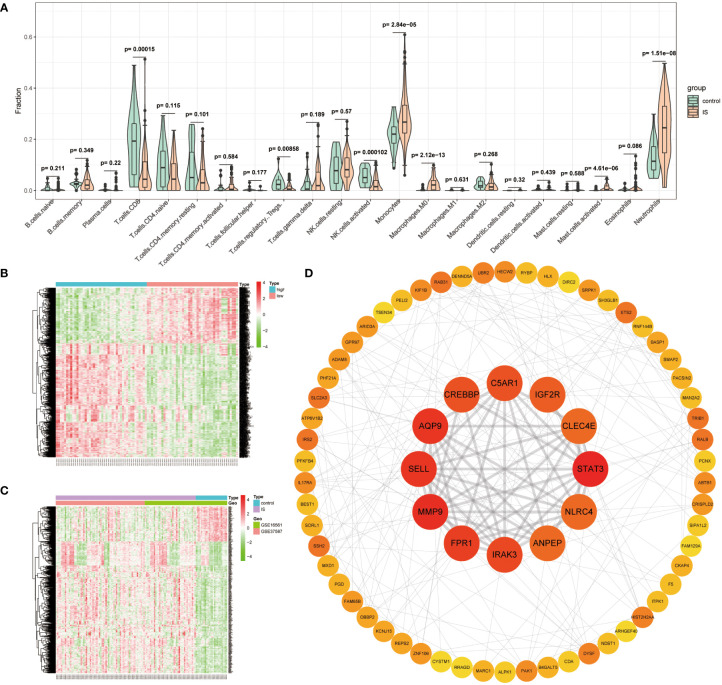
Identification of IS-specific neutrophil-related genes (ISNGS) and selection of the candidate critical genes. **(A)** Violin plot showing the difference in the infiltration proportion of 22 immune cells between IS patients and controls. **(B)** Heatmap displaying different gene expression patterns between neutrophil-high and neutrophil-low groups. **(C)** Heatmap displaying different gene expression patterns between IS and control groups. **(D)** The constructed PPI network ascertaining candidate critical genes by degree algorithm.

Since previous enrichment results confirmed that ISOSGS might play a critical role in the neutrophil response, the neutrophil distribution characteristics and its associated genes in early IS were further investigated. Specifically, we first used CIBERSORT deconvolution algorithm to estimate the fraction of 22 sorts of immune cells in each sample. Compared with healthy controls, a higher proportion for neutrophils (P = 1.51e−08) were found in IS samples (**Figure 4A**). Moreover, using the limma method, a set of 1,075 neutrophil-related DEGs were obtained between neutrophil-high and neutrophil-low group (**Figure 4B**). Similarly, we identified 559 IS-specific DEGs by comparing the transcriptome profiles of IS patients with healthy control participants (**Figure 4C**). Lastly, we intersected neutrophil-related DEGs with IS-specific DEGs and gained 402 overlapped genes defined as ISNGS, which was illustrated in the Venn diagram.

### 3.5 Identification and validation of critical genes

To identify the candidate critical genes related to both oxidative stress and neutrophil response, 72 overlapping genes by the intersection of ISOSGS and ISNGS were put into the STRING database to build a PPI network. Subsequently, through Cytoscape plugin cytohubba, 9 candidate critical genes with degree > 10 were selected, including STAT3, MMP9, AQP9, SELL, FPR1, IRAK3, CREBBP, C5AR1 and IGF2R (**Figure 4D**).

**Figure 5:**
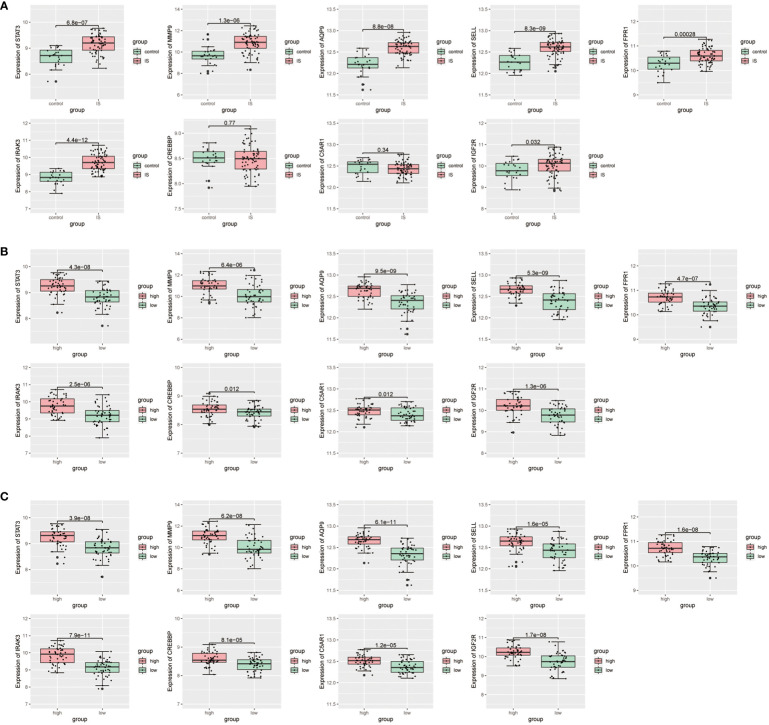
Validation for differential expression of candidate critical genes using GSE58294 dataset between **(A)** IS and control groups, **(B)** oxidative stress-high and oxidative stress-low groups, **(C)** neutrophil-high and neutrophil-low groups, respectively.

**Figure 6:**
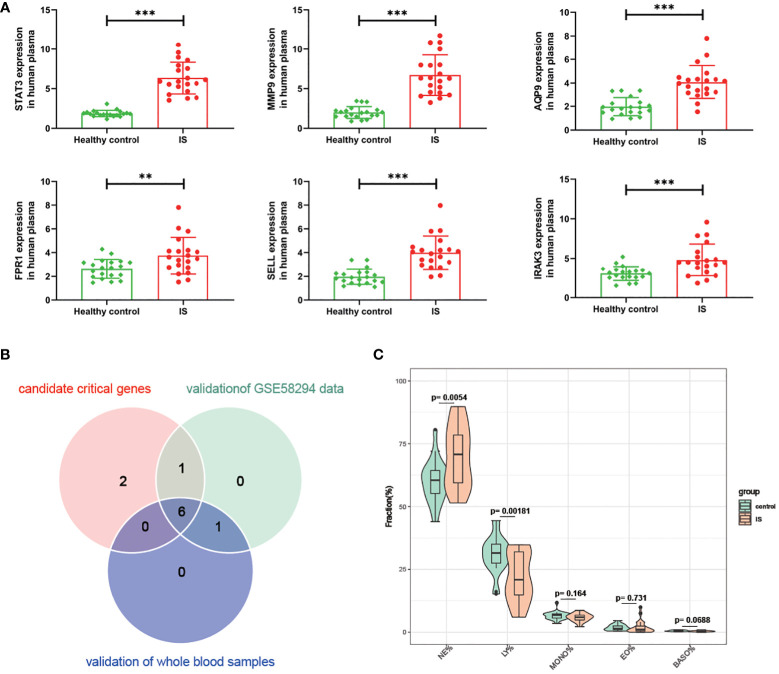
Validation for the difference of candidate critical gene expression and neutrophil percentage using clinical samples. **(A)** Verification for candidate critical genes using qRT-PCR analysis. **(B)** Venn plot of candidate critical genes and validated real critical genes. **(C)** Violin plot showing the difference of Neutrophils (NE), lymphocytes (LY), monocytes (MONO), eosinophils (EO) and basophils (BAAO) percentage in IS patients compared with controls. ** P value < 0.01 in the comparison between IS and healthy controls. ***P value < 0.001 in the comparison between IS and healthy controls.

Then, we conducted a dataset validation for the above obtained 9 candidate critical genes using the GSE58294 dataset. At the beginning of validation, the GSVA and CIBERSORT analyses were performed, the results of which again verified the up-regulation of oxidative stress status and neutrophil proportions in IS patients compared with controls (**Figures S3A**, **B**). Moreover, the expression differences of these genes between IS patients and controls, neutrophil-high and neutrophil-low groups as well as oxidative stress-high and oxidative stress-low groups were evaluated, respectively. As shown in **Figures 5A–C**, the log2-transformed expressions of 7 candidate genes, including STAT3, MMP9, AQP9, SELL, FPR1, IRAK3 and IGF2R, are significantly up-regulated in IS samples, oxidative stress-high group and neutrophil-high group. Lastly, these 7 candidate critical genes were further validated with our clinical samples by RT-qPCR method. As shown in **Figures 6A, B**, among these 7 candidate genes, 6 genes (STAT3, MMP9, AQP9, SELL, FPR1 and IRAK3) were experimentally proved to be up-regulated in the blood samples of IS patients and thus identified as real critical genes. In addition, the blood routine examination results also showed that the percentage of neutrophils in IS patients significantly increased compared with the controls, which was consistent with the trends of two CIBERSORT analysis results (**Figure 6C**).

### 3.6 Potential biological signaling pathways, diagnostic capability and predicted drugs for critical genes

**Figure 7:**
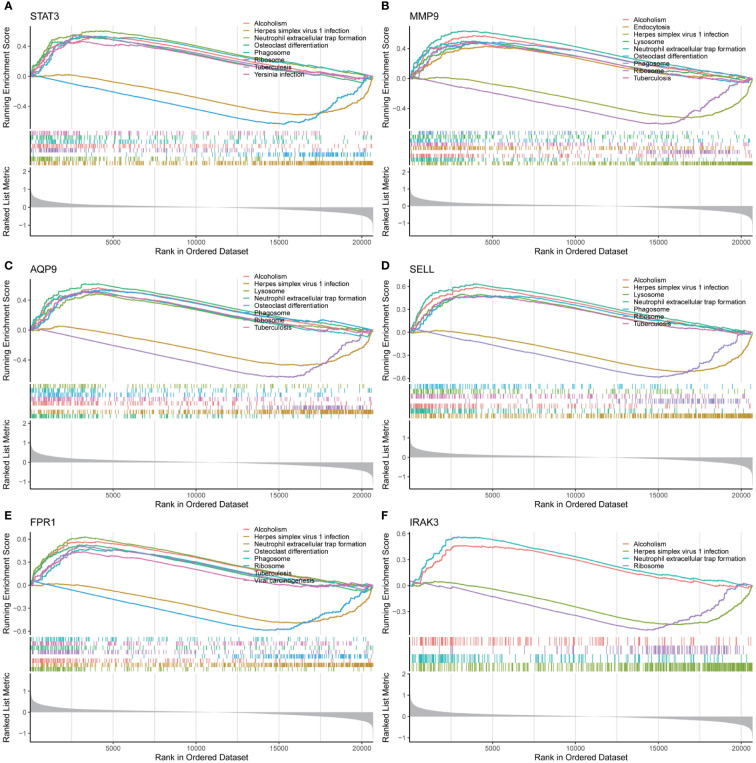
Single-gene GSEA analysis for 6 real critical genes. **(A)** STAT3, **(B)** MMP9, **(C)** AQP9, **(D)** SELL, **(E)** FPR1, **(F)** IRAK3.

To understand the important roles of these six critical genes, GSEA was applied to explore KEGG pathways that each critical gene could affect in early IS. As shown in **Figure 7**, all these six genes are involved in five signaling pathways, including neutrophil extracellular trap (NET) formation, HSV-1 infection, phagosome, ribosome, and alcoholism pathways. In addition, three critical genes (MMP9, AQP9, and SELL) may be involved in the regulation of lysosomes.

**Figure 8:**
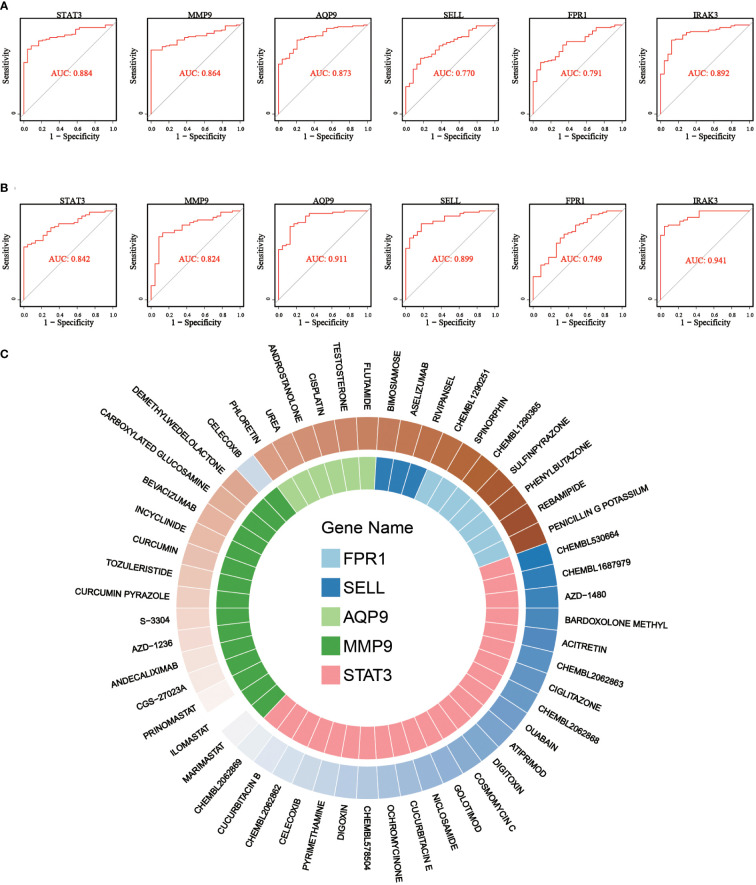
ROC curves for diagnostic performance evaluation and gene-drug interaction relationship for critical genes. **(A)** ROC curves of 6 critical genes in discovery dataset. **(B)** ROC curves of 6 critical genes in validation dataset. **(C)** Drug-gene network showing the potential interactions between 5 critical genes and 53 predicted targeted drugs.

Subsequently, the diagnostic capability of these 6 critical genes in early IS was assessed using the discovery dataset. As shown in **Figure 8A**, the AUC values of ROC curves were 0.88, 0.86, 0.87, 0.77, 0.79, and 0.89 for STAT3, MMP9, AQP9, SELL, FPR1, and IRAK3, respectively. Moreover, in the validation dataset, the AUCs of six critical genes were confirmed to be greater than 0.7 (STAT3: 0.84, MMP9: 0.82, AQP9: 0.91, SELL: 0.90, FPR1: 0.75 and IRAK3: 0.94), indicating that they had a good diagnostic performance for early IS (**Figure 8B**).

Finally, drug-gene interactions of 6 critical genes were predicted using the DGIdb database to explore potential novel drugs for early IS. After searching, we found drug-gene interactions of 5 genes except for IRAK3 and enrolled the interactions to construct a drug-gene network. As shown in **Figure 8C**, five potential gene targets (STAT3, MMP9, AQP9, SELL, and FPR1) and 53 promising drugs/ingredients constituted the drug-gene network. The obtained gene targets and drugs may provide new possibilities for the treatment of early IS and warrant further experimental study.

## 4 Discussion

Despite numerous deaths, permanent disabilities and high public burden worldwide caused by IS, the understanding of its pathogenesis and effective treatments remains limited thus far (12). Oxidative stress and inflammation, two well-known pathological mechanisms, may play crucial roles in the initiation and progression of early IS. Meanwhile, their interaction may further promote the expansion and aggravation of cerebral damage (13). Therefore, exploring the genes involved in both oxidative stress and inflammation may help uncover the novel biomarker and potential therapeutic target in the early stage of IS.

In our study, a comprehensive transcriptome bioinformatics analysis was performed to investigate critical genes related to oxidative stress and inflammation in early IS. Initially, we found ISOSGS based on GSVA and WGCNA approaches. Interestingly, their enrichment results included neutrophil degranulation, neutrophil activation involved in immune response and neutrophil extracellular trap formation, suggesting that some certain genes in ISOSGS may play pivotal roles in various neutrophil response following stroke. Next, we ascertained ISNGS adopting CIBERSORT and DEG analysis. Then, the following procedures consisting of the intersection of ISOSGS and ISNGS, PPI network construction and degree algorithm filtering, dataset and clinical samples validation were performed step by step. Eventually, six critical genes related to both oxidative stress and neutrophil response were identified (STAT3, MMP9, AQP9, SELL, FPR1, and IRAK3).

STAT3, a relatively conserved member of the STATs family, transduces signal pathways for transcriptional regulation of cellular homeostasis, proliferation, inflammation, etc (14). Previous experiments have observed that STAT3 expression levels were increased in the rat brain regions ipsilateral to middle cerebral artery occlusion (MCAO) relative to the sham group (15, 16). Moreover, Adly et al. also found elevated levels of STAT3 in the peripheral blood of patients with IS compared to controls, indicating that STAT3 may act as a vital player in the pathogenesis of IS (17). Mechanically, Agrawal et al. found that in an oxygen-glucose deprivation and reperfusion (OGD-R) model of PC12 cells, considerable reactive oxygen species (ROS) were generated, which could trigger the expression of STAT3 (18). Furthermore, in the MCAO mice, the up-regulated STAT3 could enhance IL-1β expression, thereby facilitating the recruitment and adhesion of circulating neutrophils to the damaged cerebral tissue (19–21). Then, the recruited circulating neutrophils were hyper-activated and generated more ROS and pro-inflammatory cytokines, ultimately exacerbating neuroinflammation and ischemic cerebral injury (22).

Regarding MMP9, a previous meta-analysis by Misra et al. showed that the circulating levels of MMP9 were elevated in the patients with IS and could been considered as a potential biomarker for the diagnosis of ischemic stroke (23). Moreover, a prospective observational study involving 3,186 IS patients demonstrated that the increasing levels of MMP9 in the acute phase of IS were associated with severe disability and mortality (24). Currently, the raised MMP-9 levels following IS were thought to be mainly derived from peripheral neutrophils (25, 26). Liu et al. found that ROS and reactive nitrogen species (RNS) could promote the expression and activation of MMP9 in a rat MCAO model, and thus enhance blood-brain barrier (BBB) permeability by degradation of tight junction proteins (27). Subsequently, the damaged BBB could promote more neutrophils infiltration, which in turn produced more MMP9 and ROS, eventually amplifying oxidative stress and neuroinflammation after stroke (28).

As a G protein-coupled receptor, FPR1 is distributed in various immune cells such as macrophages, monocytes, dendritic cells, and neutrophils while it has been shown to be involved in several neurological diseases, including intracerebral hemorrhage, dementia, and traumatic brain injury (29–32). Regarding IS, Li et al. observed that after 1.5 hours of MCAO and 24 hours of reperfusion, wild-type mice had larger cerebral infarct volumes and higher neurological deficit scores compared with Fpk1 knockout mice, indicating that FPR1 played a vital role in the pathogenesis of IS (33). Mechanistically, FPR1 was essential for neutrophil migration from the spleen and peripheral blood to the ischemic brain tissue, where neutrophils could exert their pro-oxidative and pro-inflammatory properties (33). On the one hand, in the neutrophils expressing FPR1, the binding of FPR1 and damage-associated molecular patterns (DAMPs) might promote extracellular influx and intracellular release of Ca2+ and subsequently cause NADPH oxidase activation by Ca2+/PKC signaling pathway, thereby generating more superoxide anion and ROS (34, 35). On the other hand, it can also accelerate the synthesis of pro-inflammatory factors including TNF-α, IL-1β, IL-6, IL-8 and MCP-1 through NF-κB pathway (36, 37).

SELL, also known as L-selectin, encodes type I transmembrane glycoprotein expressed on peripheral leucocytes with an actual molecular weight ranging from 70 to 100 kDa (38). As an adhesion molecule, it regulates the adhesion and migration of multiple immune cells and is involved in the I/R injury in a variety of organs, including the kidney and liver (39, 40). As for IS, Wei et al. recently identified SELL P213S polymorphism as a potential biomarker for IS susceptibility in the Chinese population (41). Moreover, compared with 280 healthy controls, serum SELL levels were higher in 265 IS patients, suggesting that SELL may play an important role in the occurrence and progression of IS (41). This could be explained by the following biological mechanisms. Similar to FPR1, cross-linking of SELL could activate NADPH oxidases which subsequently potentiated neutrophil oxidative burst, resulting in the generation of large amounts of ROS and more neuronal death (42, 43). In addition, with the help of PECAM-1, SELL could accelerate neutrophil migration across TNF-activated endothelial monolayers, which may facilitate circulating neutrophil infiltration into the ischemic cerebral regions (44).

As an aquaglyceroporin initially found in human circulating leukocytes, AQP9 is thought to selectively transport a variety of substances, including water, urea, etc (45, 46). In a previous experiment, Badaut et al. detected a marked increase of AQP9 levels in the mice brain following transient cerebral ischemia (47). Moreover, our study identified AQP9 in peripheral blood as a critical gene associated with early IS by bioinformatics analysis. From the perspective of mechanism, the following pathways suggest that AQP9 may play a role in the pathophysiological process of IS. For one thing, through a Rac1-dependent pathway, AQP9 was phosphorylated and relocated to the plasma membrane following fMLF and PMA activation (48). Then, AQP9 could generate a localized osmotic gradient and promote the local diffusion of polymerization-competent actin monomers by interacting with accumulated ions at the plasma membrane such as Na+, H+ and Cl- (48). Subsequently, remodeling of actin cytoskeleton led to changes in neutrophil volume and shape, ultimately facilitating trans-endothelial migration of circulating neutrophils into ischemic brain tissue (49). For another, AQP9 expressed in neutrophils could promote membrane transport of ROS, which triggered the activation of NLRP3 inflammasome (50, 51). Then, NLRP3 inflammasome could further upregulate the levels of caspase-1 and IL-1β, and exacerbate CNS inflammation and ischemic cerebral injury (51).

IRAK3, an inactive kinase of the IRAK family, is a well-known negative regulator of TLR signaling, thereby inhibiting inflammation and preventing tissue damage (52). Regarding cerebral I/R injury, Wang et al. found that activation of IRAK3 by pretreatment with TLR ligands prior to ischemia significantly prevented subsequent brain injury (53). Furthermore, Irak3 knockout mice exhibited more severe brain damage after cerebral ischemia compared with wild-type mice (54). These results indicated that IRAK3 may protect against I/R injury following IS. Mechanistically, IRAK3 inhibited the dissociation of IRAK1 and IRAK4 from MyD88 as well as their interaction with TRAF6, thus preventing the activation of NF-κB and downregulating the release of pro-inflammatory cytokines, such as IL-1β, IL-6 and TNF-α (52, 55). Besides, IRAK3 may suppress mROS production by reducing TRAF6 recruitment to mitochondria (56). Moreover, with the TLR stimulation, IRAK3 was activated and in turn negatively regulated TLR signaling, which could eventually suppress the recruitment and localization of neutrophils to ischemic brain regions (57).

As network complexes composed of chromatin DNA, histones, and granular proteins, NETs can capture and eliminate bacteria, fungi or viruses and are divided into NADPH-oxidase (Nox)-dependent NETs and Nox-independent NETs (58). Recent evidence suggested that excessive NETs could damage the host tissue in various diseases, including infection, autoimmune diseases and cardiovascular disease (59, 60). Regarding IS, our GSEA analysis identified 6 critical genes abovementioned were associated with NETs in early IS. Mechanistically, both FPR1 and SELL could regulate the activity of NADPH oxidase and thus facilitate Nox-dependent NETs generation (35, 42, 61). In addition, protein arginine deiminase 4 (PAD4) leads to Nox-independent NETs formation by increasing histone citrullination, while STAT3 could affect PAD4 expression by regulating HMGB1/TLR4 signaling pathway. In contrast, IRAK3 might block TLR4 signaling to reduce PAD4 expression (52, 61, 62). MMP9, which has been confirmed to be significantly externalized from neutrophils, may be involved in NET generation by decorating decondensed chromatin fibers released from neutrophils (63). Besides, through the ROS/NLRP3/caspase-1 pathway, AQP9 increased the expression of gasdermin-D that could puncture the plasma and nuclear membranes to release NETs (64, 65). On the one hand, intravascular NETs could induce cerebral thrombosis by providing a scaffold for platelets, red blood cells and multiple coagulation factors (66). On the other hand, NETs components from the brain parenchyma, such as histones and myeloperoxidase, could rapidly exert neurotoxicity and aggravate cerebral ischemic injury (67).

Lastly, we predicted 53 potential drugs that may exert neuroprotective effects in early stroke by targeting 5 genes (STAT3, MMP9, AQP9, SELL, FPR1). Among them, curcumin could prevent cerebral I/R damage, which could be partially explained by reducing MMP9 expression and inhibiting NETs formation (68, 69). Moreover, another three potential drugs, phloretin, cucurbitacin B, and bimosiamose, which are the inhibitors of AQP9, STAT3, and SELL, respectively, have been reported to improve neurologic deficits after rat cerebral ischemia by reducing oxidative stress (70–72). However, for most predicted drugs, especially FPR1 inhibitors, direct experimental evidence for their pharmacological effect on stroke is currently lacking. Thus, further molecular experiments are required to investigate the therapeutic effect of these predicted drugs in brain I/R injury by targeting oxidative stress and neutrophil response in early IS.

There were several merits involved in our study. To our knowledge, this was the first comprehensive transcriptome analysis to identify critical genes involved in both oxidative stress and neutrophil response and predict some potential drugs targeting these genes, which may provide new insights into the treatment of early IS. Additionally, we not only used a transcriptome-scale design, but also analyzed the data using multiple methods, including GSVA, WGCNA, and CIBERSORT, etc. Thus, our results are systematic, comprehensive, and reliable. However, some limitations should be recognized in our comprehensive analysis. First of all, studies with larger sample size are required to further validate our results, and screening pathways and genes also need to be further confirmed. Secondly, a PPI network was constructed to explore the potential biological mechanism of early IS. However, the criteria for building PPI were relatively subjective, so the results of PPI might need to be interpreted carefully. Thirdly, we systematically analyzed the roles of these six critical genes in oxidative stress and neutrophil response mainly using bioinformatics methods, and some *in vitro* and *in vivo* studies are still required to confirm their roles in early IS. Last but not least, the use of whole blood RNA analysis was the main limitation of our study, although the neutrophil response in early stroke could be detected by the transcriptome analysis using whole blood samples. As shown in the **Figure S4**, compared with controls, the neutrophil specific markers, such as ELANE, MPO and S100A8, were significantly up-regulated in IS group in the discovery dataset and our clinical whole blood samples. However, many of the target genes that are assumed to be linked with neutrophils are also highly expressed in other leukocytes. Thus, in the future, we hope to selectively extract neutrophils for further study to explore the potential crosstalk between oxidative stress and neutrophil response.

## 5 Conclusions

In conclusion, we identified 6 critical genes in early IS, including STAT3, FPR1, AQP9, SELL, MMP9 and IRAK3, that were significantly up-regulated and participated in both oxidative stress and neutrophil response, especially neutrophil extracellular trap. Our findings may provide new insights into understanding the pathogenesis mechanism and developing novel diagnostic biomarkers and therapeutic strategies for IS.

## Data availability statement

The original contributions presented in the study are included in the article/**Supplementary Material**. Further inquiries can be directed to the corresponding authors.

## Author contributions

XL and JZ conceived the study, participated in the design, and drafted the manuscript. CM, CH, and QW performed the statistical analyses, participated in the design, and helped to draft the manuscript. YW, HS, and CZ performed molecular biological studies and participated in the statistical analyses. JY, NG, and YM carried out the clinical survey and collected the samples. All authors contributed to the article and approved the submitted version.

## Supplementary material

SUPPLEMENTARY Figure S1 Data preprocessing of the discovery dataset merging two datasets. **(A)** PCA diagrams for GSE16561 and GSE37587 datasets before batch effect elimination. **(B)** PCA diagrams for IS and controls before batch effect elimination. **(C)** PCA diagrams for GSE16561 and GSE37587 datasets after batch effect elimination. **(D)** PCA diagrams for IS and controls after batch effect elimination.

SUPPLEMENTARY Figure S2 Samples clustering and determination of optimal soft threshold in the WGCNA. **(A)** Sample clustering dendrogram. **(B)** Analysis of the scale-free index for a set of soft thresholds (β). **(C)** Analysis of the mean connectivity for a set of soft thresholds.

SUPPLEMENTARY Figure S3 Validation of the oxidative stress status and neutrophil infiltration pattern in the GSE58294 dataset. **(A)** Heatmap of differential pathways showing significantly up-regulated oxidative stress pathway in early ischemic stroke. **(B)** Violin plot showing the difference in the infiltration proportion of 22 immune cells between IS patients and controls.

SUPPLEMENTARY Figure S4 Expression difference of neutrophil specific markers using discovery dataset and clinical samples. **(A)** Expression difference for neutrophil specific markers using discovery dataset. **(B)** Expression difference for neutrophil specific markers using qRT-PCR analysis.
