# Supplementary figures and images for: Crosstalk between oxidative stress and neutrophil response in early ischemic stroke: a comprehensive transcriptome analysis
Source: Front Immunol. 2023 Apr 26;14:1134956. doi: 10.3389/fimmu.2023.1134956 (PMC10169595; doi:10.3389/fimmu.2023.1134956)

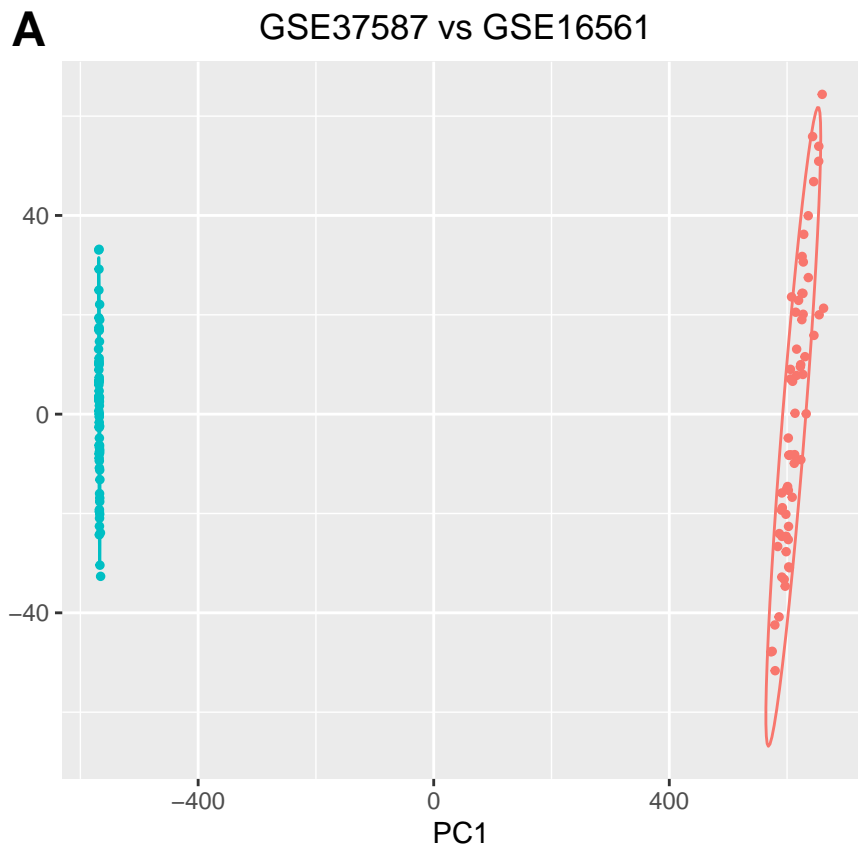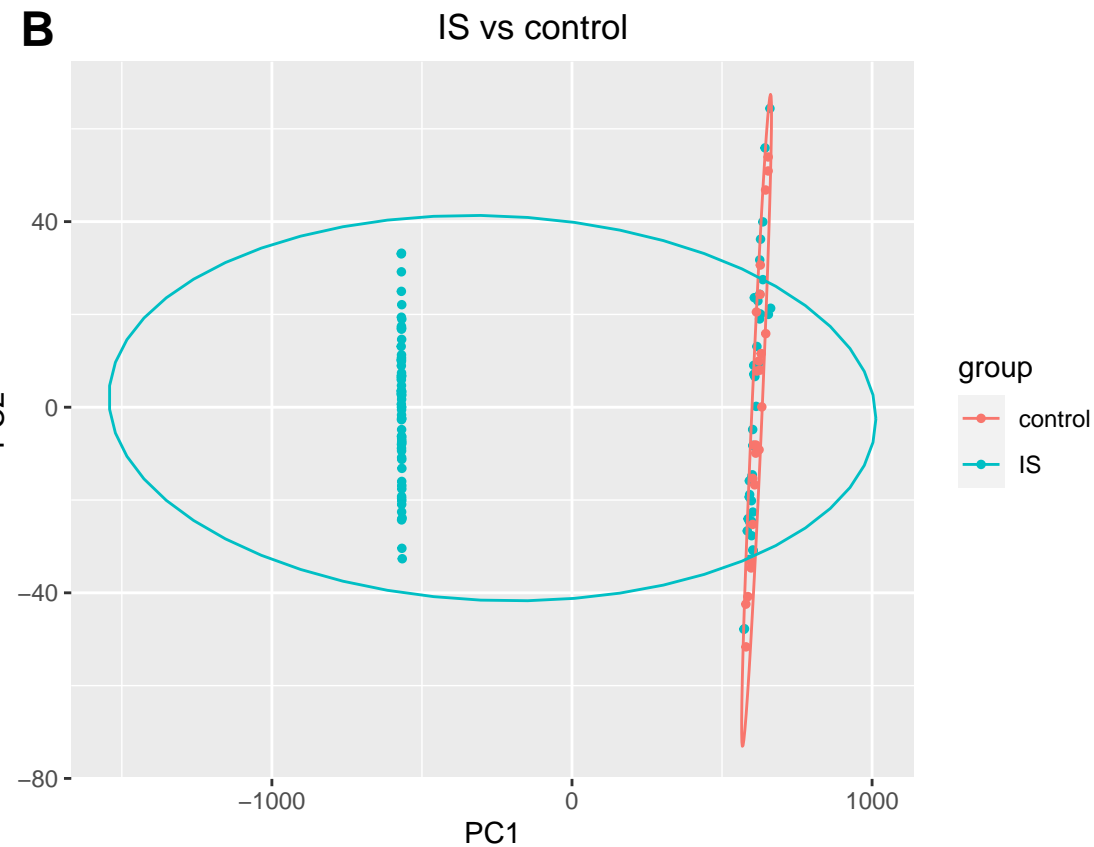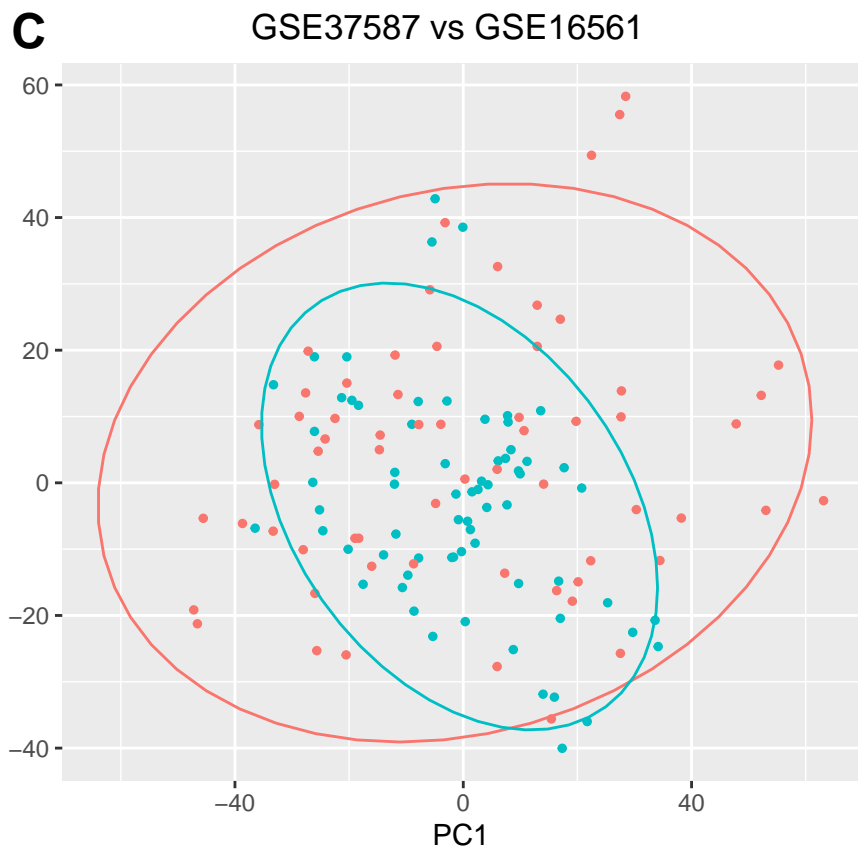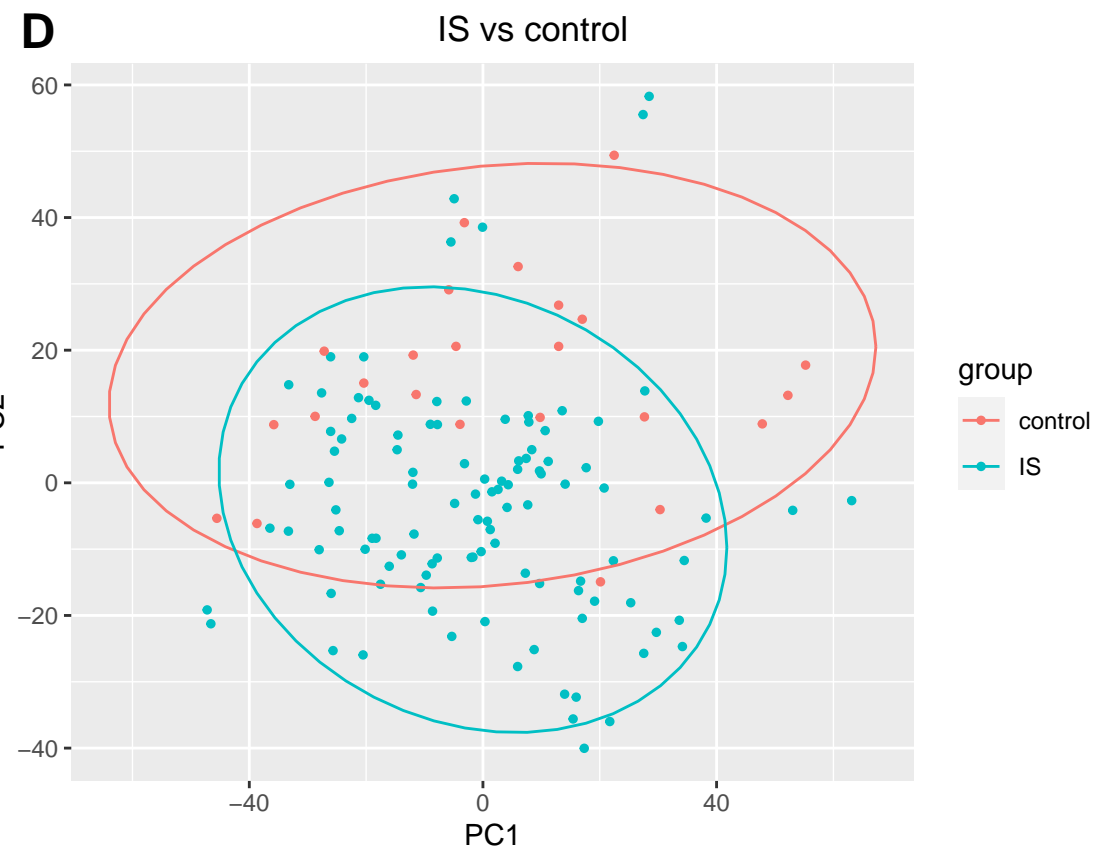

Supplement: SUPPLEMENTARY Figure S1 — Data preprocessing of the discovery dataset merging two datasets. (A) PCA diagrams for GSE16561 and GSE37587 datasets before batch effect elimination. (B) PCA diagrams for IS and controls before batch effect elimination. (C) PCA diagrams for GSE16561 and GSE37587 datasets after batch effect elimination. (D) PCA diagrams for IS and controls after batch effect elimination. [file Image_1.pdf]

A

## Sample clustering to detect outliers

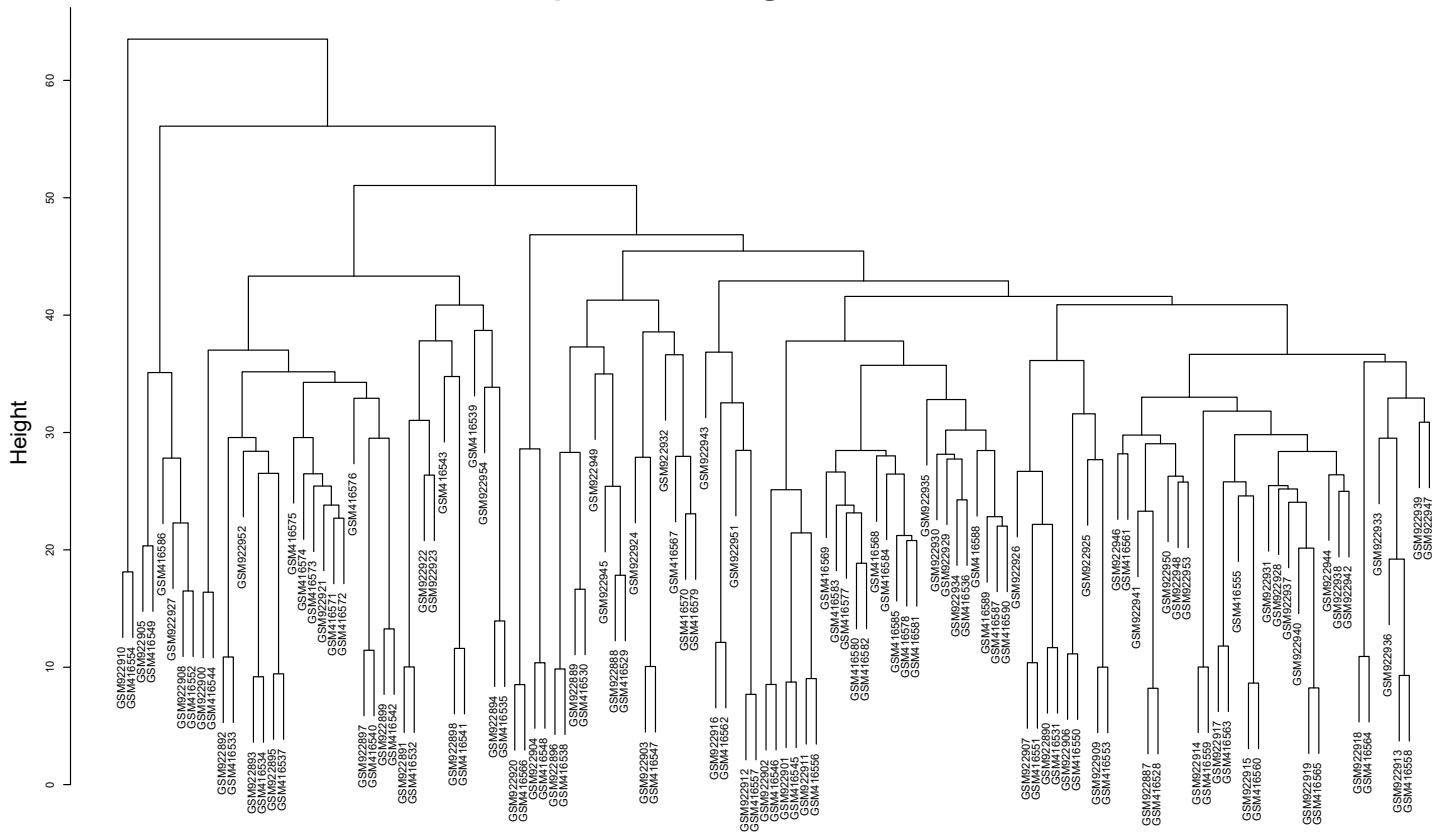

B

## Scale independence

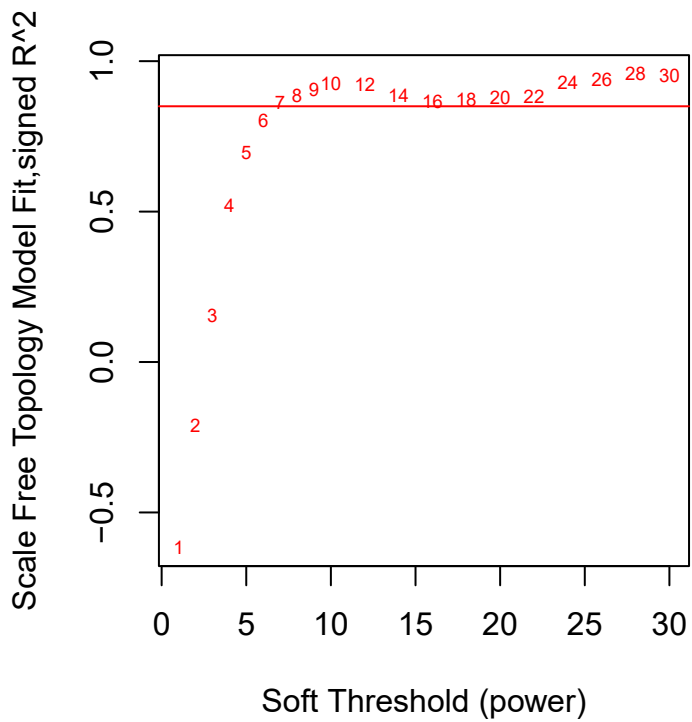

C

## Mean connectivity

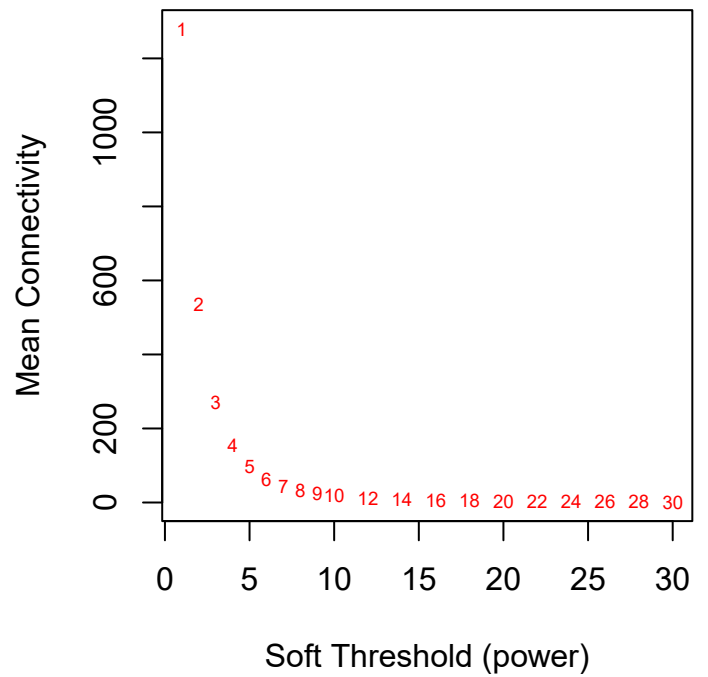

Supplement: SUPPLEMENTARY Figure S2 — Samples clustering and determination of optimal soft threshold in the WGCNA. (A) Sample clustering dendrogram. (B) Analysis of the scale-free index for a set of soft thresholds (β). (C) Analysis of the mean connectivity for a set of soft thresholds. [file Image_2.pdf]

A

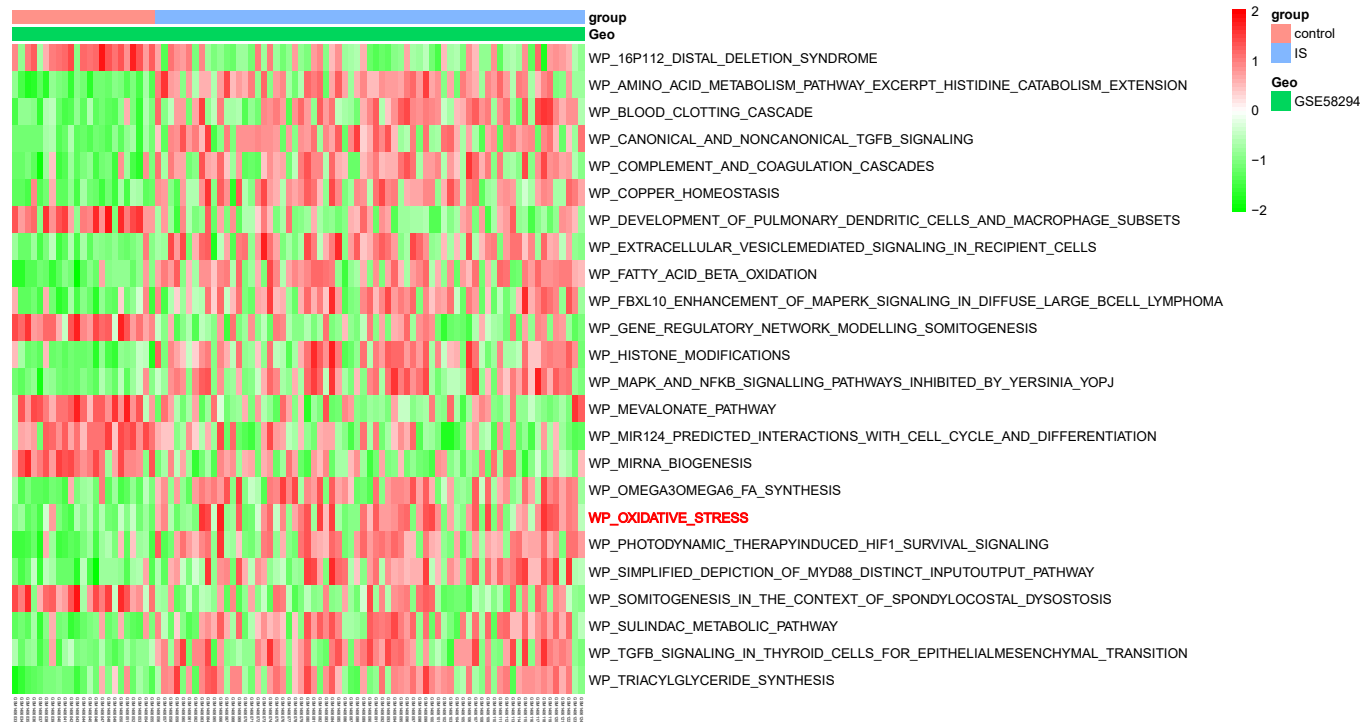

B

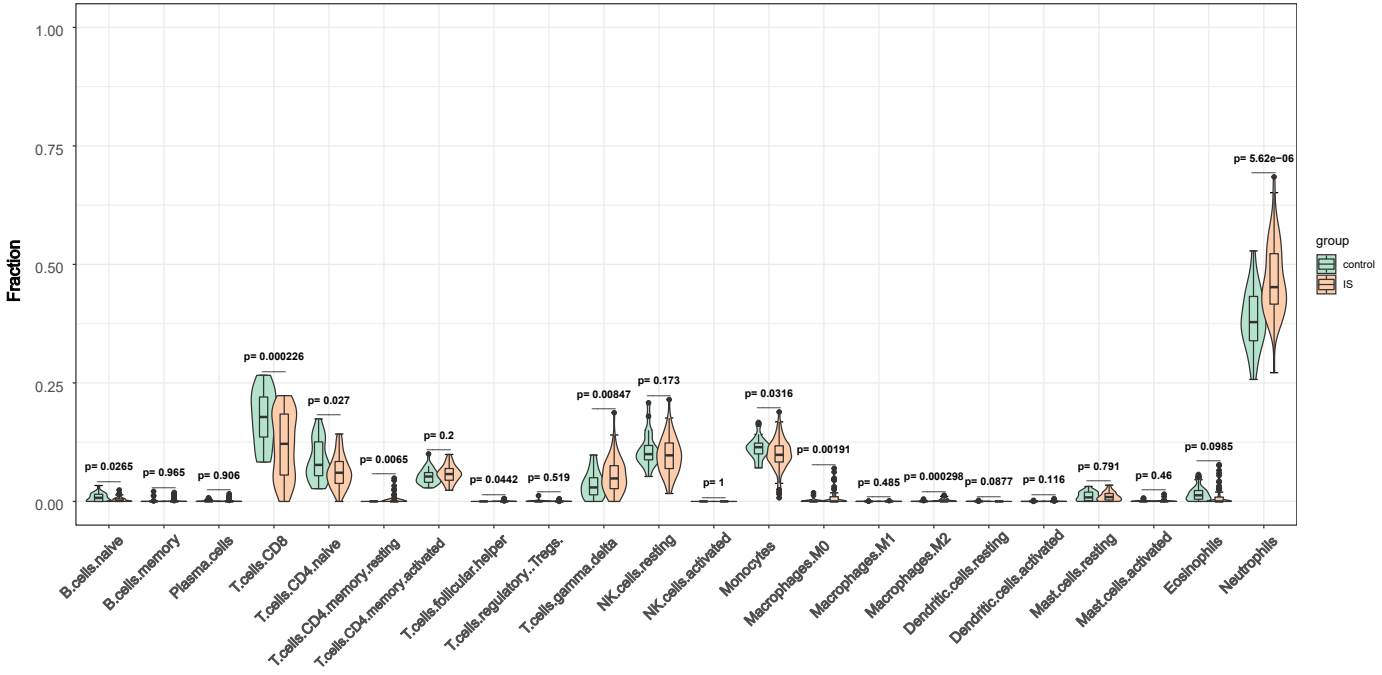

Supplement: SUPPLEMENTARY Figure S3 — Validation of the oxidative stress status and neutrophil infiltration pattern in the GSE58294 dataset. (A) Heatmap of differential pathways showing significantly up-regulated oxidative stress pathway in early ischemic stroke. (B) Violin plot showing the difference in the infiltration proportion of 22 immune cells between IS patients and controls. [file Image_3.pdf]

A

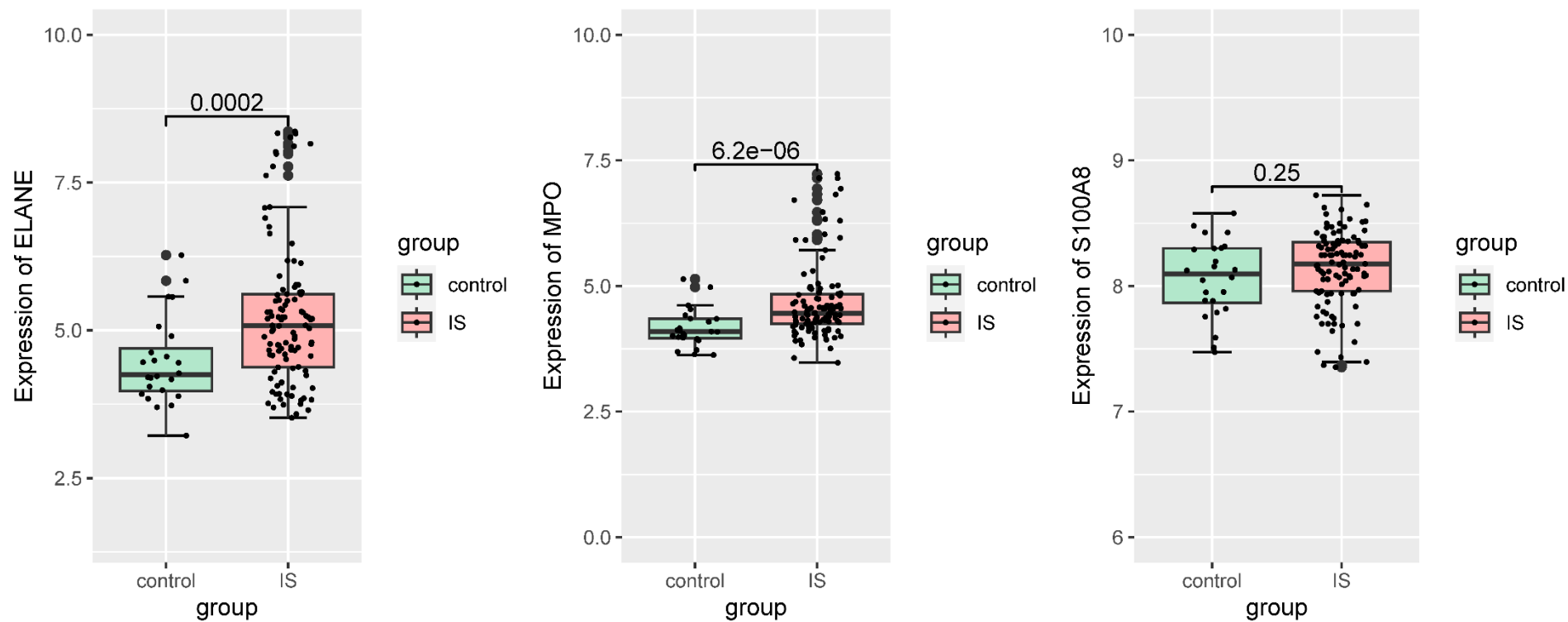

B

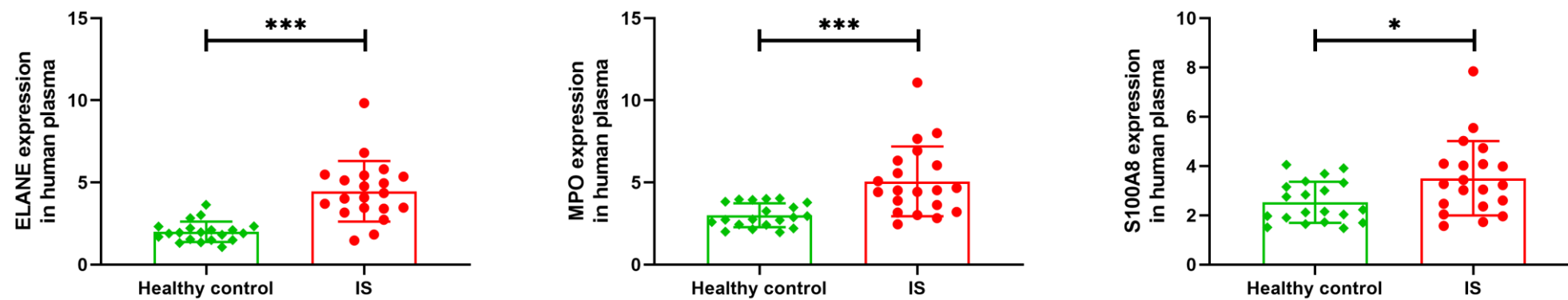

Supplement: SUPPLEMENTARY Figure S4 — Expression difference of neutrophil specific markers using discovery dataset and clinical samples. (A) Expression difference for neutrophil specific markers using discovery dataset. (B) Expression difference for neutrophil specific markers using qRT-PCR analysis. [file Image_4.pdf]
