# Supplementary material for: Crosstalk between oxidative stress and neutrophil response in early ischemic stroke: a comprehensive transcriptome analysis
Source: Front Immunol. 2023 Apr 26;14:1134956. doi: 10.3389/fimmu.2023.1134956 (PMC10169595; doi:10.3389/fimmu.2023.1134956)
Supplement: Supplementary file 5 [file Table_1.docx]

**Table S1: Basic information on the enrolled gene expression profiles**

| GEO Accession | Platform | Samples | Collection method  (Storage conditions) | Number of cases | Number of controls | Time collection of cases | Author (year) |
| --- | --- | --- | --- | --- | --- | --- | --- |
| Discovery dataset |  |  |  |  |  |  |  |
| GSE37587 | GPL 6883 | whole blood samples | Paxgene blood RNA tubes* | 34 IS | 0 controls | 0-24h after onset of IS (N=34) | Barr (2015) |
|  |  |  | (-80℃ freezer) |  |  | 24-48h after onset of IS (N=34) |  |
| GSE16561 | GPL 6883 | whole blood samples | Paxgene blood RNA tubes | 39 IS | 24 controls | 0-24h after onset of IS (N=39) | Barr (2010) |
| Validation dataset |  |  | (-80℃ freezer) |  |  |  |  |
| GSE58294 | GPL570 | whole blood samples | Paxgene blood RNA tubes | 23 IS | 23 controls | 0-3h after onset of IS (N=23) | Stamova (2014) |
|  |  |  | (unavailable) |  |  | 5h after onset of IS (N=23) |  |
|  |  |  |  |  |  | 24h after onset of IS (N=23) |  |

IS: ischemic stroke, *: PAXgene blood RNA Tubes contain a proprietary reagent composition based on a patented RNA stabilization technology. This reagent composition protects RNA molecules from degradation by RNases.
