# Supplementary material for: Crosstalk between oxidative stress and neutrophil response in early ischemic stroke: a comprehensive transcriptome analysis
Source: Front Immunol. 2023 Apr 26;14:1134956. doi: 10.3389/fimmu.2023.1134956 (PMC10169595; doi:10.3389/fimmu.2023.1134956)
Supplement: Supplementary file 6 [file Table_2.docx]

**Table S2. Clinical characteristics of validation samples.**

| Variable | IS Group | Control Group | *P* Value |
| --- | --- | --- | --- |
| Age | 54.9± 13.7 | 59.7 ± 12.2 | 0.24 |
| Male | 15 (75%) | 13 (65%) | 0.49 |
| BMI | 23.9 ± 3.6 | 24.6 ± 2.9 | 0.97 |
| Hypertension | 12 (60%) | 8 (40%) | 0.21 |
| Diabetes | 7 (35%) | 4 (20%) | 0.29 |
| Hyperlipidemia | 7 (35%) | 6 (30%) | 0.74 |
| Smoking | 4 (20%) | 3 (15%) | 1 |
| Drinking | 5 (25%) | 3 (15%) | 0.69 |

BMI: body mass index
