# Supplementary material for: Crosstalk between oxidative stress and neutrophil response in early ischemic stroke: a comprehensive transcriptome analysis
Source: Front Immunol. 2023 Apr 26;14:1134956. doi: 10.3389/fimmu.2023.1134956 (PMC10169595; doi:10.3389/fimmu.2023.1134956)
Supplement: Supplementary file 7 [file Table_3.docx]

**Table S3. Sequences of primers used for qPCR.**

| Gene |  | Sequence |
| --- | --- | --- |
| STAT3 | forward | 5’-CACCAAGCGAGGACTGAGCATC-3’ |
|  | reverse | 5’-AGCCAGACCCAGAAGGAGAAGC-3’ |
| MMP9 | forward | 5’-AGTCCACCCTTGTGCTCTTCCC-3’ |
|  | reverse | 5’-TCTCTGCCACCCGAGTGTAACC-3’ |
| AQP9 | forward | 5’-GGTGTCTCTGGTGGTCACATCAAC-3’ |
|  | reverse | 5’-CCCACAAAGGCTCCCAAGAACTG-3’ |
| SELL | forward | 5’-ACAAGGAGGACTGCGTGGAGATC-3’ |
|  | reverse | 5’-TTAGTTTGTGGCAGGCGTCATCG-3’ |
| FPR1 | forward | 5’-GCTGTATCTGCTGGCTATCTCTTCC-3’ |
|  | reverse | 5’-GGTAACTGATGGTGGTGACTGTGTG-3’ |
| IRAK3 | forward | 5’-GCGGGCAAAGTTAAGACCATCAATG-3’ |
|  | reverse | 5’-AGAGGAGAAGGACACCTGAAGGAC-3’ |
| IGF2R | forward | 5’-GGAGGCTGTAAGGACGGAGGAG-3’ |
|  | reverse | 5’-GGAGGGCAACGATCACCATTCAC-3’ |
| GAPDH | forward | 5’-GTCTCCTCTGACTTCAACAGCG-3’ |
|  | reverse | 5’-ACCACCCTGTTGCTGTAGCCAA-3’ |
